# Patients’ and healthcare providers’ perceptions and experiences of telehealth use and online health information use in chronic disease management for older patients with chronic obstructive pulmonary disease: a qualitative study

**DOI:** 10.1186/s12877-021-02702-z

**Published:** 2022-01-03

**Authors:** Yuyu Jiang, Pingping Sun, Zhongyi Chen, Jianlan Guo, Shanshan Wang, Fenglan Liu, Jinping Li

**Affiliations:** 1 Research office of chronic disease management and rehabilitation, Wuxi School of Medicine, Jiangnan University, No. 1800 Lihu Avenue, Wuxi, 214122 Jiangsu Province China; 2 Department of Nursing, Wuxi School of Medicine, Jiangnan University, Wuxi, 214122 Jiangsu Province China; 3 School of Medicine, Liaocheng University, Liaocheng, 252000 Shandong Province China; 4 Department of Public Health, Wuxi School of Medicine, Jiangnan University, Wuxi, 214122 Jiangsu Province China

**Keywords:** Older people, Telehealth, eHealth literacy, Chronic disease management, Online health information, Qualitative study, Chronic obstructive pulmonary disease, Healthcare providers

## Abstract

**Background:**

Telehealth and online health information provide patients with increased access to healthcare services and health information in chronic disease management of older patients with chronic diseases, addressing the challenge of inadequate health resources and promoting active and informed participation of older patients in chronic disease management. There are few qualitative studies on the application of telehealth and online health information to chronic disease management in older patients. Chronic obstructive pulmonary disease is one of the most common chronic diseases in older adults. Telehealth is widely used in the management of chronic obstructive pulmonary disease. The purpose of this study was to explore the perceptions and experiences of older patients and healthcare providers in the application of telehealth and online health information to chronic disease management of chronic obstructive pulmonary disease.

**Methods:**

A qualitative descriptive study with data generated from 52 individual semi-structured interviews with 29 patients [Law of the People’s Republic of China on the protection of the rights and interests of older people (2018 Revised Version) = >60 years old] with chronic obstructive pulmonary disease and 23 healthcare providers. The inductive thematic analysis method was used for data analysis.

**Results:**

Four themes and 16 sub-themes were identified in this study. Four themes included: faced with a vast amount of online health information, essential competencies and personality traits ensuring older patients’ participation and sustained use, user experience with the use of technology, being in a complex social context.

**Conclusion:**

The ability of patients to understand health information should be fully considered while facilitating access to online health information for older patients. The role of health responsibility and user experience in older patients’ participation and sustained use of telehealth and online health information needs to be emphasised. In addition, the complex social context is a determining factor to be considered, particularly the complex impact of a reliance on offspring and social prejudice on the behaviour of older adults using telehealth and online health information.

**Supplementary Information:**

The online version contains supplementary material available at 10.1186/s12877-021-02702-z.

## Background

The rapid ageing of the world’s population has made chronic disease management (CDM) in older people a major challenge in public health [1–3]. However, many factors, such as the insufficient and unequal distribution of health resources and cost issues, prevent older people from accessing CDM [4–6]. Telehealth, which refers to the use of information and communication technologies to deliver healthcare services, facilitates interactions between patients and healthcare providers (HCPs) at a distance [7]. Telemedicine, telehealth education and remote rehabilitation via smartphone applications or dedicated websites are all telehealth interventions [8–11]. Due to the openness and interactive functions of the internet and the availability of a massive amount of information online, people can also access health-related information using various online channels, such as search engines, health web portals and social media [12]. We refer to this type of health-related information as online health information. Telehealth interventions and online health information have the advantages of saving health resources, regardless of time and space, and being economical and convenient [7, 9, 13–15]. Telehealth use and online health information use in CDM can address the abovementioned barriers [7, 9, 13–15]. Furthermore, in the trend towards a patient-centred model of healthcare delivery, the use of telehealth and online health information guarantees more active and informed patients in CDM [16–20].

According to Norman et al. [21] and Griebel [22], eHealth literacy encompasses the ability to seek, find, understand, appraise, communicate, create health information from electronic resources and apply the acquired knowledge to address or solve health problems. Older adults with chronic conditions who have adequate levels of eHealth literacy are able to participate in and benefit from telehealth interventions and online health information. Previous surveys suggested that older adults generally had low levels of eHealth literacy [23, 24], and that they had barriers to participating in telehealth interventions and online health information [25]. Some older adults with chronic diseases and HCPs were found to remain negative about the application of telehealth [26]. Researchers have highlighted that productive interactions between patients with chronic conditions and HCPs are key to CDM [27]. There is a need to explore patients’ and HCPs’ perceptions and experiences of the application of telehealth and online health information to CDM in older patients.

Qualitative research on telehealth applications in CDM has focused on exploring consumers’ needs and potential barriers [28, 29], as well as experiences and perceptions of using a particular website/smartphone application or participating in a particular telehealth intervention programme (e.g., video conferencing, remote monitoring) [30–32]. Qualitative studies of online health information are few in number and mostly take a single perspective. Most of these studies have focused on eHealth literacy and have been designed to explore only consumers’ perceptions and experiences of seeking, finding, understanding, appraising, and applying online health information [33, 34]. There are few qualitative studies of patient and HCP perspectives on older adults engaging with telehealth interventions and online health information in CDM [35, 36].

Chronic obstructive pulmonary disease (COPD) is one of the most common chronic diseases of older adults. Telehealth intervention programmes have been developed for the self-management of COPD, pulmonary rehabilitation and other CDM programmes for COPD. Some findings have suggested that such telehealth programmes can improve quality of life and reduce hospitalisation rates in patients with COPD [9, 35–37]. However, researchers have noted that the implementation of some telehealth intervention programmes was difficult due to technical difficulties, low patient compliance and lack of personalisation [38]. The purpose of this study was to explore the perceptions and experiences of older patients and HCPs in the application of telehealth and online health information for CDM of COPD. It was expected to provide guidance and experience in improving the eHealth literacy of older adults and promoting engagement with telehealth interventions and online health information among older adults, so that the advantages of telehealth use and online health information use in CDM for older adults could be exploited.

## Methods

### Study design

A qualitative descriptive study was conducted using individual semi-structured interviews to gather information. This study was performed in accordance with the Declaration of Helsinki and was approved by the Medical Ethics Committee of Jiangnan University (JNU20190318IRB61).

### Study participants and settings

A purposive sampling method was used to recruit participants. Patients were recruited from local hospitals and COPD management groups. HCPs were recruited from local hospitals and health care teams delivering pulmonary rehabilitation through Pulmonary Internet Explorer Rehabilitation (PeR, mobile technology, a free social media WeChat official account). All participants were recruited directly by the researchers of this study.

When recruiting patients, we tried to recruit individuals with a range of ages, genders, education levels, disease classifications and disease durations. Patients were divided into three groups according to the following: those who were recruited from local hospitals and attended only routine follow-up visits for COPD were categorised as the regular group, those who participated in pulmonary rehabilitation through PeR were categorised as the PeR group, and those who participated in pulmonary rehabilitation delivered face-to-face were categorised as the FtF group. Eligibility criteria for older patients with COPD included the following: (1) age of 60 years or older [39], (2) confirmed diagnosis of COPD according to the diagnosis and treatment guidelines for COPD [40], forced expiratory volume in 1 s (FEV1)/forced vital capacity (FVC) < 0.7, (3) ability to communicate in Chinese regardless of speaking speed [41], and (4) no mental illness related problems during the study.

When recruiting HCPs, we tried to recruit individuals with a range of genders and working durations. HCPs who carried out pulmonary rehabilitation interventions for older COPD patients through PeR were grouped into the PeR group. HCPs recruited from local hospitals were grouped together. HCPs involved in this study only included doctors, nurses and physical therapists (PTs). HCPs were expected to have experience having been involved in the diagnosis and treatment/rehabilitation/care of patients with COPD.

The patients in the PeR group and the FtF group in this study were from the intervention group and control group, respectively, of a randomised controlled trial (RCT) (ChiCTR1900022770) [9]. The HCPs in the PeR group in this study provided a pulmonary rehabilitation intervention through PeR for older COPD patients in the RCT. Interviews with these patients and HCPs took place post-intervention. In the RCT, patients in the intervention group received a 3-month rehabilitation training (including breathing exercises, exercise training, dietary guidance, medication knowledge, etc.) through PeR and uploaded training records and self-assessment reports on PeR. They reported training records at least once a week and had access to health professionals in the event of acute exacerbations. HCPs gave real-time feedback based on patient reports. Without the use of PeR, patients in the control group received the same rehabilitation intervention training face to face in the outpatient clinic.

The 54 eligible participants were contacted either face-to-face or by phone due to the limitations imposed by the COVID-19 pandemic. These participants included 31 patients and 23 HCPs. The researchers explained the study to them in both oral and text forms. Participants had 1 week to consider participation in the interviews and decide whether to accept or refuse. The two patients who refused to participate in the interviews reported that they knew nothing about this topic. The 52 participants who accepted the interviews, including 29 patients and 23 HCPs, provided informed consent and participated voluntarily.

### Data collection

Self-designed questionnaires were used to collect general information about participants. Based on the concept of eHealth literacy, separate interview guides were developed for older patients with COPD and HCPs (see Additional file 1), which were piloted among 2 patients and 2 HCPs. There was no revision needed. The data from these pilot interviews were included in the analysis.

Both the first author (J) and the second author (S) were female, and they conducted interviews. Field notes were recorded during the interviews. The first author (J) is a teacher with rich experience in qualitative research and quantitative research, focusing on pulmonary rehabilitation and telehealth for CDM. With 4 years of learning and practical experience in qualitative research, the second author (S) is a postgraduate student majoring in geriatric nursing. The interviewers made an effort to establish rapport with the participants to communicate effectively with them and to obtain authentic and rich data. Face-to-face interviews were conducted pre-COVID in a quiet reception room at the medical school of Jiangnan University and telephone interviews were conducted with participants at home during COVID. No third person was present during any of the interviews. This helped ensure a safe environment in which participants could speak freely.

Participant recruitment, data collection and data analysis were carried out simultaneously. After 52 interviews, no new themes were developed, indicating that data saturation had been reached, and we stopped the interviews. All interviews were audio-recorded. The average duration of the interviews was approximately 30 min (18–75 min).

### Data analysis

The inductive thematic analysis method was used for data analysis [42]. In this study, Word (software) was used to manage qualitative data and tables in Word were used to carry out data analysis. Within 24 h after each interview, the recorded data were transcribed in Chinese verbatim by the interviewers (J and S). They reviewed the transcripts to check for accuracy and returned them to participants for confirmation. In addition, they (J and S) read transcripts repeatedly to familiarize themselves with the transcripts. The authors (J and S) read the transcripts independently, identified the key quotes, and performed the initial coding. Members of the research team met regularly to discuss and review the codes, eventually reaching consensus on the codes. Collating these agreed codes and their anchor statements, one author (S) continuously compared the similarities and differences between these codes, assigning the codes to potential themes/sub-themes, namely, initial themes. The other author (J) reviewed these themes and the codes under the themes. These two authors(J and S) discussed identifying the similarities and differences among the codes and the themes. Themes were developed with codes combined and categorised. An advisory group contributed to the interpretation of the findings. The advisory group comprised of nine people, including three patients (PeR = 1, FtF = 1, Regular = 1), two doctors (PeR = 1, non-PeR = 1), two nurses (PeR = 1, non-PeR = 1) and two PTs (PeR = 1, non-PeR = 1). They supported the analysis by providing feedback on the meaning ascribed to the findings. After discussion and review among team members, four final themes were developed: faced with a vast amount of online health information, essential competencies and personality traits ensuring older patients’ participation and sustained use, user experience with the use of technology, and being in a complex social context.

The quotes from the results were translated into English by a translation team consisting of one member of the research team and two people outside the research team who were proficient in English. One researcher is a returned scholar, who checked the translated text and provided feedback to the translation team with questions until agreement was reached.

## Results

### General information

The characteristics of the 29 older patients with COPD and 23 HCPs interviewed are summarised in Table 1 and Table 2. Thirteen patients (PeR = 5, FtF = 4, Regular = 4) were interviewed by telephone, and 16 patients (PeR = 5, FtF = 6, Regular = 5) were interviewed face-to-face. Eight HCPs (Doctor = 2, Nurse = 5, PT = 1) were interviewed by telephone, and 15 HCPs (Doctor = 6, Nurse = 6, PT = 3) were interviewed face to face.

**Table 1:** Demographics of older patients with COPD (*n* = 29)

Characteristics	P ^a^ (*n* = 29)
Gender, n (%)	
Male	19 (66)
Female	10 (34)
Age (years), mean (SD)	70.6 (6.4)
Education status, n (%)	
Primary school and Below	7 (24)
Middle school	10 (34)
High school	8 (28)
Higher education and Above	4 (14)
Disease classification ^b^, n (%)	
GOLD I	5 (17)
GOLD II	11 (38)
GOLD III	6 (21)
GOLD IV	7 (24)
Disease duration, n (%)	
< 10 years	12 (41)
≥ 10 years	17 (59)
Group	
PeR group ^c^	10 (34)
FtF group ^d^	10 (34)
Regular group ^e^	9 (31)

^a^ *P* patient

^b^ *GOLD* Global Initiative for Chronic Obstructive Lung Disease; GOLD I: FEV1/FVC < 70%, FEV1 ≥ 80%; GOLD II: FEV1/FVC < 70%, 50% ≤ FEV1 < 80%; GOLD III: FEV1/FVC < 70%, 30% ≤ FEV1 < 50%; GOLD IV: FEV1/FVC < 70%, FEV1 < 30%

^c^ PeR group: Older patients with COPD who participated in CDM for COPD through Pulmonary Internet Explorer Rehabilitation (PeR, mobile technology, a free social media WeChat official account) belong to PeR group

^d^ FtF group: Older patients with COPD who participated in CDM for COPD face-to-face belong to FtF group

^e^ Regular group: Older patients with COPD who participated in routine follow-up visits for COPD belong to regular group

**Table 2:** Demographics of HCPs (*n* = 23)

Characteristics	D ^a^ (*n* = 8)	N ^b^ (*n* = 11)	PT ^c^ (*n* = 4)
Age (years), mean (SD)	44.4 (8.0)	32.5 (5.0)	41.5 (10.5)
Gender, n (%)			
Male	6 (75)	0	2 (50)
Female	2 (25)	11 (100)	2 (50)
Degree, n (%)			
Bachelor	5 (63)	8 (73)	3 (75)
MSc ^d^	2 (25)	3 (27)	1 (25)
MD ^e^	1 (13)	0	0
Working duration, n (%)			
< 10 years	2 (25)	5 (45)	1 (25)
≥ 10 years	6 (75)	6 (55)	3 (75)
Position, n (%)			
Head	5 (63)	3 (27)	2 (50)
None	3 (38)	8 (73)	2 (50)
Training experiences			
Yes	5 (63)	6 (55)	4 (100)
No	3 (38)	5 (45)	0
Experience with PeR ^f^			
Yes	4 (50)	6 (55)	2 (50)
No	4 (50)	5 (45)	2 (50)

^a^ *D* doctor

^b^ *N* nurse

^c^ *PT* physical therapist

^d^ *MSc* Master of Science

^e^ *MD* Doctor of Medicine

^f^ *PeR* Pulmonary Internet Explorer Rehabilitation

### Main results

Four themes and 16 sub-themes were identified in this study (Table 3).

**Table 3:** Themes and sub-themes

Themes	Sub-themes
1. Faced with a vast amount of online health information	1.1 Health-related information that older patients contemplated 1.2 The understanding of health-related information available to older patients
2. Essential competencies and personality traits ensuring older patients’ participation and sustained use	2.1 Basic personal competencies of older patients for accessing telehealth and online health information 2.2 Confidence of older patients 2.3 Personal inertia of older patients 2.4 Health responsibility of older patients
3. User experience with the use of technology	3.1 Novel feeling of technology in older patients 3.2 Feeling superior to other older people 3.3 Feeling of distance between the patient and HCP during remote interaction 3.4 Users’ scepticism of the accuracy of remote diagnosis 3.5 Perceived advantages of using telehealth and online health information by users
4. Being in a complex social context	4.1 Social prejudice against the internet 4.2 People’s resistance to the telehealth service model 4.3 The reliance on offspring 4.4 The persuasive power of speech 4.5 Health resources to be improved

### Faced with a vast amount of online health information

Faced with a vast amount of online health information, older patients had reasons for accessing online health information and contemplated the content of health information. Participants reported that older patients’ understanding of the health information available to them influenced their health behaviours.

### Health-related information that older patients contemplated

Most patients and HCPs reported that knowledge of chronic disease, knowledge of traditional Chinese medicine (TCM) related to health promotion, information about seeking medical services, and major outbreak information were types of health information that older COPD patients contemplated. Older patients often searched for the above information online. “Wonder drugs” were a particular health information concern for older people with COPD.

*“COPD is really my concern … I gasp even getting dressed. I often search to see if there is any good solution or ‘wonder drugs’. I also asked the COPD patients in WeChat group what medicines work ” (P6, PeR)*

*“We Chinese people care a lot about health maintenance. People my age should go online to learn more about health care, such as acupressure... This is good both for ourselves and our families” (P22, Regular)*

*“If I sensed an illness, I would definitely baidu (a google-liked Chinese search engine) which hospital is the best for the treatment and rehabilitation of it ... and what’s the most conveniently way to get there ” (P21, Regular)*

*“Everything COVID-related is my biggest concern now ... for example how to properly wear a mask.” (P21, Regular)*

*“Mostly I go online to see if there is a ‘wonder medicine’ for COPD” (P25, Regular)*

Some patients reported that a lack of information led them to search for COPD-related health information online.

*“I wanted to know what to pay attention to in general (for COPD patients). At the hospital, the doctors were too busy to talk about these stuff. I had to look it up on the internet myself. It was said that tai chi could be good for COPD, so I have tried it.” (P24, Regular)*

However, a minority of patients reported that frequent exposure to negative disease-related online news caused their own psychological discomfort and they were reluctant to access disease-related health information online.

*“I don't want to go online because a lot of people there say that COPD is a disease that can't be cured … negative views like that make me feel uncomfortable.” (P13, FtF)*

### The understanding of health-related information available to older patients

Some older patients reported that they were able to distinguish health information available online and apply the knowledge.

*“I knew something about COPD like it can’t be cured. So when I saw some medicine claiming to cure COPD, I knew it’s a lie. ” (P26, Regular)*

*“There is a lot of useful health information online ...... I went online and found out that people with chronic obstructive pulmonary disease should get a flu shot every year to prevent colds. I get a flu shot every year and it is working” (P7, PeR)*

However, some older patients were more likely to “take online information out of context” and made health decisions that were detrimental to their own health. Some HCPs argued that this tendency interfered with the delivery of their health services.

*“I have searched online ... Although some said smoking is bad, others said it has benefits ... so I choose to stay with smoking” (P25, Regular)*

*“Some older people misunderstood the information on the internet ... making our work more difficult” (D3)*

In addition, a small number of patients felt overwhelmed by the amount of information available. They reported that they were unable to distinguish what information was useful to them and gave up searching online and employing online health information.

*“Information or services about COPD rehabilitation are available on WeChat, Today’s Headline and television ... It’s just too much. I don't know how to choose or which one to use. So I just quit and let the doctor decide.” (P23, Regular)*

### Essential competencies and personality traits ensuring older patients’ participation and sustained use

Participants talked about some of the basic personal competencies that enabled older patients to use telehealth and online health information. Participants also talked about some of the personality traits (inertia, confidence, responsibility) ensuring older patients’ engagement and sustained use of telehealth and online health information.

### Basic personal competencies of older patients for accessing telehealth and online health information

Most participants agreed that a certain level of vision, literacy, level of Mandarin and learning and memory skills were essential for older patients to engage with telehealth interventions and online health information.

Patients and HCPs reported poor access to text-based online health information for older patients with poor vision.

*“I’m too old to recognize the small size words on my cell phone” (P14, FtF)*

Patients and HCPs described that older patients’ lack of literacy skills limited their ability to access written materials and communicate health information through text. Along the same lines, older patients with insufficient levels of Mandarin had difficulties in accessing audio materials and vocally communicating health information.

*“Some older patients did not even finish primary school. They can't read... write or type. It's hard for them to communicate with health care professionals through cell phone ... Most of the videos on health education for COPD are in Mandarin … they only speak the local dialect and do not understand Mandarin. ” (N1)*

Patients and HCPs also noted that some older patients with poor learning and memory skills struggled to quickly grasp how to use smart devices and software when engaging with telehealth interventions and online health information.

*“We taught the older patients to use PeR to participate in chronic disease management for COPD ...some of them are slow to learn and quick to forget” (N8, PeR)*

### Confidence of older patients

Participants described older patients’ beliefs in being able to learn, engage and sustainably participate in telehealth use and online health information use.

Both older patients and HCPs noted that most older COPD patients lacked confidence and were intimidated when learning telehealth, tending to give up learning and/or trying to use telehealth and online health information.

*“I'm afraid I can’t master it (to use a cell phone) ... so I quit. ” (P17, FtF)*

*“Some older patients found using PeR too difficult for them … gave up before they even started” (N6, PeR)*

Older patients and HCPs reported that experiences of success or failure with learning and/or using telehealth determined patients’ confidence and willingness to continue learning and participating in telehealth use and online health information use.

*“I’ve learnt it before, for example, the play button... I’ve been following* PeR *for pulmonary rehabilitation before so I’ m experienced. I wanted to find breathing exercises, and found it within a few clicks.” (P5, PeR)*

*“I was not proficient with cell phone before. I was afraid I couldn't do it ... Now I can follow the videos on PeR to exercise on my own. I think I can master it … it’s a good tool and I will use it more often.” (P6, PeR)*

*“At first I was quite willing to learn. But after I learnt (how to search for health information online), I forgot it within a few days. So I learnt it again. I went back and forth for several times. It felt like I couldn't remember a thing and I couldn't learn. I lost my confidence and simply gave up.” (P28, Regular)*

### Personal inertia of older patients

Some patients and HCPs noted that some older patients were reluctant to access or did not access telehealth and online health information because of personal inertia.

*“I haven't been paying much attention to rehabilitation information related to COPD on the Internet lately ... Sometimes when I'm just too lazy for that.” (P12, FtF)*

*“Some older people have inertia ... they are just too lazy to learn or look it up online” (N3)*

### Health responsibility for older patients

Some patients believed that they should take responsibility for their own health. They reported regular and proactive use of the internet for health information related to CDM for COPD and the application of self-management information related to COPD.

*“My view is that we should be responsible for our own health, we can't rely on others completely ... should rely on ourselves. I can always go online to find out what I want to know and get advice. Now I do the breathing exercises I found on the internet every day” (P20, FtF)*

However, some older patients reported not having enough time and energy to participate in telehealth interventions and go online to access online health information.

*“I'm usually too busy looking after my granddaughter and cooking … to have enough energy. How could I have extra time to search health information online? “ (P15, FtF)*

A few patients reported that they would forward online health information to family members and close friends to maintain the health of others. However, there were also patients who were reluctant to forward health information to other patients online because they feared that their forwarded information would damage others’ health, especially health information concerning medications.

*“I forwarded some useful health information to good friends and family members ... that’s good for their health” (P26, Regular)*

*“I simply read but did not forward it. There are truths and lies on the internet. Forwarding health information to others may lead to unwanted results. Who should be responsible if their conditions worsen?” (P5, PeR)*

### User experience with the use of technology

Participants talked about their subjective experiences of using and their expectations of using technology products that enabled telehealth interventions and online health information, such as smartphones, portals, and applications.

### Novel feeling of technology in older patients

Older patients and HCPs reported that some older patients found telehealth to be a novelty. These patients were interested in telehealth and were willing to try accessing online health information and participating in remote CDM.

*“It's new and quite interesting. I'd love to try ... to search for information on common medications for COPD online. I like to follow the guidance of PeR to do rehabilitation exercises. Isn't that great?” (P5, PeR)*

*“They found it novel ... happy to participate in the chronic disease management of COPD with PeR “ (N5, PeR)*

### Feeling superior to other older people

Older patients mentioned that the act of being able to independently search for health information online and access telehealth services made them feel superior to their peers.

*“ I always keep up with the trends. When they [peers] saw that I could consult the doctor online on what to do about COPD, they would say ‘You’re so fashionable! This is awesome! Could you teach me?’ They wanted to be like me.” (P21, Regular)*

*“My friends said I was good at this (using telehealth) … asked me to help him find hospitals and buy medicine online ... I think I am better than them on this matter” (P16, FtF)*

### Feeling of distance between the patient and HCP during remote interaction

Older patients and HCPs reported insufficient psychological support for remote interactions compared to that in face-to-face interactions for communication and consultation between older patients and HCPs and reported that patients were less inclined to access healthcare services via smart devices.

*“Smart devices are not people … not human enough to replace us ” (N9, PeR)*

*“Spacing out through cell phone makes people feel distant from each other … better to communicate in person” (P23, Regular)*

*“It's not just the exchange of disease information ... eye-contact and a gesture from a doctor or a nurse will give the patient a lot of psychological comfort, which is very difficult to achieve on a mobile phone.” (D4)*

### Users’ scepticism of the accuracy of remote diagnosis

Some older patients were sceptical about the accuracy of the results of teleconsultation and were reluctant to apply telehealth during disease consultations.

*“How can doctors conduct a follow-up consultation online? ...without listening by a stethoscope and tapping by fingers. Just saying a few words ... without a physical examination, there must be something wrong with the diagnosis” (P12, FtF)*

*“Looking, listening, questioning and feeling the pulse are important in Traditional Chinese Medicine. If doctors don't see the patient, the diagnosis must have been faulty “ (P24, Regular)*

HCPs also felt that there were problems with the accuracy of remote consultations.

*“I think online consultations are more limited ... are not as accurate as face-to-face consultations” (D4)*

### Perceived advantages of using telehealth and online health information by users

Patients and HCPs noted that some patients were willing to learn and engage with telehealth and online health information because they felt they were easy to use and convenient.

*“The system and device (for telehealth) must be simple and practical. The older person will only learn to use it if they find it’s easy to learn” (D2)*

“*...notes on COPD ... more convenient to look up online.*” (P24, *Regular*)

*“I don't have to go to Shanghai if I want to see a specialist ... I can consult a doctor at home anytime through PeR without going to the hospital. I think this form of telehealth is great and I will continue to use it in the future” (P2, PeR)*

HCPs also reported that they recommended telehealth to patients because of its economic cost and lack of potential for cross-infection.

*“The cost of transport for patients to visit doctors is reduced with remote communication ... no risk of cross-infection. We would like to recommend it to patients” (D8, PeR)*

Both patients and HCPs also noted that COVID-19 pandemic advanced the use of telehealth among older people with COPD.

*“During the pandemic, it was very inconvenient for older patients to go to the hospital. We created a WeChat group to facilitate COPD patients' consultation and communication. Many older patients were very active in the group ... asking about information on diet, medication ...” (N4)*

### Being in a complex social context

The social environment is complex due to social moral values, individuals’ social support systems, and health resources. Participants recognised that older patients’ participation in telehealth interventions and online health information in the management of chronic conditions was influenced by this complex social environment and produced outcomes of a certain complexity.

### Social prejudice against the internet

Participants generally felt that accessing health information or healthcare services online was prone to problems such as online fraud, privacy breaches and adoption of incorrect information leading to health damage. Some older patients refused or abandoned telehealth applications. Some older patients were more cautious about using online health information and participating in telehealth services. They often adopted strategies such as engaging in repeated searches and seeking verification from HCPs.

*“There are too many scammers online … often scamming money through fake health management platforms. So I won't try it.” (P29, R)*

*“I don't go for online rehab exercises ... a few clicks might give your privacy away.” (P13, FtF)*

*“Online rehab exercises for COPD ... I would consider whether it would be good for my health. If so, I would like to follow it ... If online health information said I needed to take medication, I would be more cautious… looking it up on the internet to check or asking my doctor. What if I took the wrong medication and messed up my body” (P19, FtF)*

*“Some older patients came to me and asked if they could take a medicine that they learned about online” (N3)*

Patients and HCPs emphasised that better regulation of information by government departments was the only way to change people’s prejudices about the internet.

*“Unless it is regulated by the government … only this can prevent fraud and information leakage. Then people can trust the internet” (P29, Regualr)*

*“It’s up to the relevant departments of the state to do a good job of standardization.” (N6, PeR)*

### People’s resistance to the telehealth service model

Participants generally perceived telehealth as a new model of healthcare delivery. They felt that it took time for people to accept something new. The conservative mentality of older people and the working mindset and process habits of HCPs prevented telehealth from being adopted overnight.

*“We all know that telehealth is the future” (P5, PeR)*

*“We all know for accepting the change in the healthcare model hardly to happen in a short term ... A shift of mindset is even harder. We have become accustomed to the original face-to-face delivery of health care ... It takes time to change our work mindset and procedure “ (D6, PeR)*

*“We all know that older people are relatively conservative … identify with tradition … find it difficult to change … to accept new models of healthcare.” (D1)*

Some patients also expressed a feeling of being forced to use telehealth and were concerned that the traditional model of healthcare delivery would be completely replaced by telehealth in the future.

*“If telehealth is as common as e-payment, I’ll have to use it even if I don't like it” (P11, FtF)*

*“I am really scared that I will have to use telehealth in the future” (P27, Regular)*

Some HCPs believed that primary care and telemedicine services are complementary. However, there were also HCPs who believed that telemedicine services are not needed if there are well-established primary care services.

*“Primary care services and telemedicine services should be complementary to each other.” (PT2, PeR)*

*“Is there a need for internet healthcare to exist? Now the country is vigorously developing primary care and the hierarchical diagnosis and treatment system. If the community health service centres can solve all the patients' problems, why do we need online medical care?” (D2)*

### The reliance on offspring

Influenced by traditional Chinese culture (children should practice filial piety with their parents and should care for and support their parents) and negative stereotypes of older people (the widespread belief that older patients have poor self-care skills and learning and memorizing ability, as well as low proficiency in using electronic products), older patients themselves, their peers, and HCPs rightfully viewed older patients’ offspring as responsible for ensuring the patient’s health and acting as the patient’s preferred supporter and assistant during the use of telehealth services. (P21, Regular) (P19, FtF) (D4) (D8, PeR) (N1).

Some older patients reported that the support and assistance of their offspring was a sign of their offspring’s filial piety and they felt happy about this. (P21, R) In addition, HCPs felt that the involvement of patients’ offspring could improve the efficiency and effectiveness of remote CDM for older COPD patients, as well as reduce their work stress and avoid potential risks of their liability. (D4) (D8, PeR) (N1)

*“Searching, consulting doctors ... No matter what difficulty I met, my son would teach me. My son is very filial, which makes me very happy … many friends envy me.” (P21, R)*

*“ It is their sons and daughters who should be teaching them. ‘Raising a child for old age’. If they want to learn, their children should teach them” (P19, FtF)*

*“There is no point in talking too much to older patients. In China, older adults always discuss things with their children, and they rely on their children to maintain their rights and health. In fact, by teaching their children, things (remote CDM for COPD) will be twice as easy, and older patients can just ask their children for help when they have problems” (D4)*

*“It is known that older people are slow learners … have poor memory and are not as skilled as younger people in using electronic devices. If they want to participate in remote CDM, they definitely need someone to help. Their children are definitely the first choice ... On-site training for patients and families on how to use PeR ... to ensure the effect of CDM … and to reduce our work pressure.” (D8, PeR)*

*“ It is known that older patients have poor self-care skills and need their children to accompany and look after them ... Their children are the target group for health education. Videos are usually for their family members … and communication in person for patients “ (N1)*

Patients and HCPs reported that some older patients were dependent on their children and did not practice telehealth themselves. (P23, R) Some older patients reported their experiences of learning and trying to use telehealth by themselves with the guidance and assistance of their children. (P21, R) Some patients experienced lack of help from their children. (P20, FtF)

*“I didn’t consult my doctor online … my daughter did it all. I didn’t have to learn and use it myself” (P23, R)*

*“They (children) are always in a hurry. My son taught me several times, but he spoke too fast for me to follow” (P20, FtF)*

### The persuasive power of speech

Patients and HCPs felt that patient and slow speech, easy-to-understand explanations, and a friendly voice and tones made older patients more willing to learn about and use telehealth.

*“The young lady (doctor) didn't talk fast. What she said was easy to understand. She spoke in a fine voice like my granddaughter. I was just willing to listen to and follow what she said (… participating in CDM for COPD using PeR)” (P9, PeR)*

*“The person in my ward was also an older patient with COPD. He told me how he searched the internet for information about COPD … clearly and vividly … as if he was telling a story … easy to understand. I liked to learn from him” (P18, FtF)*

The use of rhetorical devices (such as analogies, proverbs), motivational or praising words, assertions and repetition was reported to enhance the expressive effect of speech and to reinforce the patient’s self-confidence in learning and participating in telehealth.

*“‘It’s never too old to learn.’ was how we all encouraged him” (N7, PeR)*

*“‘You are not that old ... PeR is much easier than playing mahjong. It's a piece of cake for you’. The doctor describing it in such a humorous way made me at ease. It's no big deal … just try to learn and use it ” (P6, PeR)*

*“The older people should be told ‘you can do it!’ repeatedly and emphatically. Compliment the person after he/she has done it. The older people will then have the confidence to apply PeR well to manage their illness.” (N9, PeR)*

Both older patients and HCPs noted that older patients were more likely to follow the advice of trusted HCPs or listen to the instructive language of authoritative HCPs to use telehealth.

*“The APP (the telehealth CDM APP) recommended by director in the Respiratory Department must be good. I trust the doctor who work in tertiary hospitals. I will use it for sure.” (P10, PeR)*

*“I have done exercises on PeR almost every day, because the doctor emphasized ‘you must follow PeR exercises and make sure to practice at least 3 days a week’ ” (P8, PeR)*

Some HCPs suggested that HCPs be trained in conversational speech before carrying out remote CDM.

*“Healthcare professionals should also be trained in a uniform way before carrying out remote chronic disease management … it is crucial to train people on wording and making medical terminology easy to understand.” (N9, PeR)*

### Health resources to be improved

Most HCPs felt that they did not have sufficient time and energy to participate and offer timely feedback in telehealth consultation and medical services, leading to a lower satisfaction rate among older patients.

*“The amount of time I devoted to telehealth can be described as a drop in the bucket. I was too busy with my work and life, and I didn’t have extra energy ... to give timely feedback to patients’ online questions. So patients’ experience may not be as good and levels of satisfaction not as high” (D2)*

HCPs pointed out that the motivation of HCPs to use telehealth was linked to incentives.

*“In order to promote telehealth services for health care professionals … People will be motivated if there is a moderate pay rise … or a higher possibility of promotion for the telehealth work they perform.” (D2)*

HCPs emphasised that the charge system, legislation and regulations, and matching information systems for telehealth needed to be improved.

*“We should develop matching software information systems to support the development of telehealth ... develop and improve laws and regulations to address medical liability issues. Such telehealth services should not be free of charge … the corresponding pricing system and health care payment system should be updated” (D6, PeR)*

## Discussion

This study explored the experiences and perceptions of telehealth use and online health information use in CDM in older people with COPD from the perspective of patients and HCPs. Several studies have investigated user (patient/carer and staff) perspectives and experiences of telehealth in COPD management [43]. In terms of the underlying competencies and personality traits that ensure patient engagement and sustained use, Williams et al. found that “patients’ transition from being uncertain about their ability to use the technology to being confident to use it” during the use of mHealth apps to support COPD self-management [30], which is similar to the findings of this study. With regard to user experience regarding the use of technology, several studies have suggested that the usability of equipment, convenience, and greater accessibility were perceived by patients and/or HCPs as benefits of telecare or tele-monitoring [44–46]. HCPs also focused on the potential economic benefits of telehealth in reducing healthcare costs [45]. Ure et al. found that clinicians were concerned about false positive symptom scores in the implementation of COPD tele-monitoring services [46]. These findings are similar to the findings in this study regarding users’ scepticism of the accuracy of remote diagnosis and users’ perceived advantages of using telehealth and online health information. Concerning the complex social context, Ure et al. and MacNeill et al. found that HCPs believed or feared that telehealth led to an increased workload [46, 47], and this was also mentioned as part of the subtheme “health resources to be improved” in this study. In addition, some findings identified in this study are reflected in existing eHealth literacy models, such as the findings related to health-related information that older patients contemplated, the understanding of health-related information available to older patients, basic personal competencies of older patients for accessing telehealth and online health information, confidence of older patients, and perceived advantages of using telehealth and online health information by users [21, 48–55]. The new findings from this study included those related to personal inertia, health responsibility, feeling superior to other older people, a feeling of distance between the patient and HCP during remote interaction, reliance on offspring and the persuasive power of speech.

Health literacy is defined as “the degree to which individuals have the capacity to obtain, process, and understand basic health information and services needed to make appropriate health decisions” [56]. In addition to well-known health information of concern to patients, such as, knowledge of chronic disease and information on seeking medical services [28, 33], this study found that information about “wonder drugs” and TCM related to health promotion was a particular concern of older patients when they searched online. This study suggested that online health information can bridge the divide in the amount of health information available to individuals. However, the ability of older patients to make appropriate health decisions is closely related to their ability to understand this health information. Studies have suggested that health literacy levels among older people are generally low [57, 58]. Understanding health information can be challenging for older people with no medical background. Therefore, it is not useful to simply enhance an individual’s skills in searching for information on computers or smartphones. When conducting telehealth interventions, it is recommended that the aforementioned health information that older patients contemplated, such as health information from culturally specific traditional medicine, be provided in an easy-to-understand manner for older patients. In particular, HCPs should take care to correct patients’ misconceptions about wonder drugs in their daily consultations/care/health education. A number of health communication strategies have been demonstrated to help individuals understand health information [59, 60]. For example, the teach-back method prompts patients to “show” the provider the information being communicated (repeat her or his understanding of the information back to the HCPs) and to receive clarifying feedback, which is a non-stigmatising way of enhancing and confirming the patient’s understanding [61, 62]. The “clear communication” strategy suggests using plain language, focusing on 1–3 key messages, repeating or summarising key messages and suggestions, and using multiple teaching channels (e.g., visual aids and written summaries) [60, 63].

Individuals have the responsibility to change their health behaviours to maintain physical health, mental health and social health [64, 65]. Researchers have noted that promoting personal health responsibility can improve health behaviours and promote chronic disease self-management in older patients [65, 66]. This could explain the finding of this study that patients with a high level of personal health responsibility proactively practice telehealth and adhere to healthy behaviours. Notably, this study found for the first time that older patients refused to forward health information to others because they feared that their forwarded information would damage others’ health. In terms of the above phenomenon, researchers can try the following two strategies to develop eHealth literacy interventions and promote patients’ telehealth application. First, researchers can intervene in personal health responsibility to encourage older patients to practice telehealth, which can also improve patients’ ability to screen and appraise online health information. Second, researchers can encourage patients to forward health information and communicate with others by emphasising that such behaviour reflects a moral character to help others [67]. There is little research on the above view, which should become a research point in future. The importance of personal health responsibility is also included in the connotation of healthy ageing [64, 65, 68]. Thus, promoting personal health responsibility among older people not only is important in eHealth literacy interventions but also is in line with the World Health Organization (WHO) ’s concept of healthy ageing.

This study showed that there were a number of advantages to telehealth. However, the lack of inspection, palpation, percussion and auscultation, or the “four measures of TCM diagnosis”, made patients and HCPs sceptical of the accuracy of a telehealth diagnosis. During telehealth interactions, the lack of nonverbal emotional support made patients and HCPs feel distant. Therefore, both the telehealth service style and tele-technology need to be optimised. First, hybrid online and offline healthcare services can meet different patients’ needs. Rianne et al. also pointed out that the application of blended care is becoming a trend [69]. Its initial effects, such as improving the quality and efficiency of care, increasing patient and provider satisfaction with services, and improving patient health outcomes, have been proven [70–73]. Furthermore, with the development of artificial intelligence technology and its application in telehealth, the diagnostic accuracy of inspection, palpation, percussion and auscultation during telehealth will be improved. In addition, during telehealth interaction between HCPs and older patients, the feeling of distance can be relieved by a high level of emotional interaction technologies.

Zimbardo [74] pointed out that individual behaviour could be influenced in contexts such as interpersonal, persuasive and mass media, which is known as social influence. An empirical study confirmed that perceived risk mediated the impact of social influence on behavioural intentions, further influencing actual online participation [75]. Some online safety issues, such as fake information or misinformation, have become prevalent, and fake information and misinformation are widely disseminated by social media and people [76, 77]. When consumers adopt new technologies, the sharing of such information about online safety issues by social media and those around them can cause negative cognitive responses (such as perceived risks) [78], leading consumers to refuse to use online health information. That point is consistent with the findings of this study. Several meta-analysis studies have suggested that the quality of online health information is problematic [79, 80]. Previous studies found that older people were more likely to disseminate false online health information [81], and that the adoption of false information could lead to “unhealthy behaviours” [82]. Therefore, while consumers are encouraged to access health information online, measures need to be taken to enhance consumers’ skills in distinguishing the authenticity of online information effectively. For example, consumers should be clearly informed about the professionalism of online health information providers, and consumer media literacy can be enhanced through the News Literacy Project and the MediaLit toolkit from the Media Literacy Centre [77].

The findings in this study suggested that in persuading and guiding people’s participation in telehealth interventions and online health information, a number of key elements reinforced the role of speech, such as the matching of voice and intonation and the use of rhetorical devices. The findings of this study were supported by the previous researchers’ emphasis on the importance of phrasing skills and nonverbal signals/paralanguage (e.g., vocal tones and expressions) during communication [74, 83]. Some researchers have noted that expertise accounts for only 15–20% of communicator credibility, while concern and empathy account for approximately 50%. Empathy can be judged in the first 9–30 s of meeting from the mental aura of the communicator (including dress, hair, posture and facial expressions) [83]. During verbal communication, the matching of voice and intonation not only helps create emotional resonance between the communicators, but also facilitates the educational content accepted and internalized. Several studies and experiments have also demonstrated that paralinguistic persuasion attempts (i.e., modulating the voice for persuasion) can be effective in influencing perceivers’ attitudes and choices during the persuasion process [84]. The literatures have reported that rhetorical devices make speech more appealing, enhance its expressive effect, guarantee the effective transmission of semantic messages and improve the effectiveness of persuasion and education [83, 85]. Zimbardo suggested that group hints and “call and response” also had the effect of persuading and guiding, but the application of these techniques in older patients has yet to be clinically validated [74]. Therefore, during telehealth CDM, it is important to pay attention to conversational speech training in communicators to make full use of the persuasive power of speech, achieving a persuasive and guiding effect on patient behaviour and improving efficiency.

This study found that both older people and HCPs are dependent on the offspring of older patients. This has some relevance to traditional Chinese culture. As a result of traditional Chinese Confucian emphasis on filial piety and the concept of “raising children for old age”, it is taken for granted that the children of older patients are patients’ dependents. Older patients’ children also calmly accept HCPs’ and their parents’ reliance on them. Traditional culture varies among different nations. Regarding how children should treat their parents, Chinese Confucianism emphasises “filial piety” in terms of respecting, obeying, caring and supporting one’s parents, while the Western Bible emphasises “honour” in terms of respecting one’s parents. The role of filial piety in the phenomenon of the reliance on offspring in different cultural contexts during participation in telehealth interventions and online health information remains to be elucidated. Therefore, cultural characteristics should be considered when telehealth CDM or eHealth literacy interventions are developed. This study demonstrated the advantages of moderate reliance on offspring in remote CDM. Previous studies also confirmed that family involvement in CDM can improve patient quality of life and adherence. Such evidence has been practised by HCPs [86, 87]. However, this study also suggested that overreliance on offspring hindered older patients from practising telehealth. Therefore, when telehealth CDM is conducted, it is necessary to assess the degree of reliance of older patients on their offspring and help with the implementation of individualised interventions. In this way, older patients should practice telehealth and benefit from telehealth CDM. There is a lack of assessment tools to assess the degree of reliance of older people on their offspring. Some attachment theories and scales in psychology could help researchers develop tools [88, 89]. If we look at the phenomenon of overreliance on offspring dialectically, we believe that overreliance on offspring can be a solution to some extent when the CDM service system and service capacity cannot meet the requirements of older patients. However, this can only be used as a temporary measure in some areas.

### Implications

To maximise the benefits of telehealth interventions and online health information in the CDM of older COPD patients, the joint efforts of older patients and their offspring, HCPs, technology developers, policy makers and media practitioners are needed.

In the process of integrating telehealth into existing healthcare services, policy makers need to consider the complementary nature of telehealth services and traditional face-to-face health services in healthcare services and their different scope of application. Policy makers also need to pay attention to the rational allocation of human health resources and the improvement of appropriate health systems and regulations. As far as online health information is concerned, there is a need for greater regulation by the relevant national authorities. At the same time, the media should pay attention to correctly guiding the public’s attitude towards online health information, encouraging active participation and prudent treatment. In this way, a favourable environment can be created for the management of chronic diseases in older patients.

In the development and implementation of telehealth programmes, the level of health literacy and emotional support needs of older people should be fully considered. The persuasive and guiding role of HCPs and older people’s peers should be brought into play. Moderate participation of older people’s children should be encouraged and guided.

### Limitations

There were several limitations to this study. First, the sample of the study covered only the main participants in CDM for COPD in China, without the inclusion of other people, such as family members of older patients and information engineers. Researchers have identified patients, family members or caregivers, information engineers, and policy makers as important stakeholders in the development and implementation of telehealth. To promote the uptake and impact of telehealth programmes, the views and suggestions of these stakeholders should be fully considered [22, 90]. In addition, this study could not cover all telehealth management programmes for COPD.

## Conclusions

The ability of patients to understand health information should be fully considered while facilitating access to online health information for older patients. The role of health responsibility and user experience in older people’s participation and sustained use of telehealth and online health information needs to be emphasised. In addition, the complex social context is a determining factor to be considered, particularly the complex impact of a reliance on offspring and social prejudice on the behaviour of older adults using telehealth and online health information.

## Supplementary Information

**Additional file 1.** Interview guides for older patients with COPD and for HCPs.

## Data Availability

The datasets used and/or analysed during this study are available from the corresponding author on reasonable request.

## Abbreviations

CDM: Chronic disease management
HCPs: Healthcare providers
COPD: Chronic obstructive pulmonary disease
FEV1: Forced expiratory volume in 1 s
FVC: Forced vital capacity
PTs: Physical therapists
RCT: Randomised controlled trial
GOLD: Global Initiative for Chronic Obstructive Lung Disease
TCM: Traditional Chinese medicine
WHO: World Health Organization
COREQ: Consolidated criteria for reporting qualitative research
