# Supplementary material for: Patients’ and healthcare providers’ perceptions and experiences of telehealth use and online health information use in chronic disease management for older patients with chronic obstructive pulmonary disease: a qualitative study
Source: BMC Geriatr. 2022 Jan 3;22:9. doi: 10.1186/s12877-021-02702-z (PMC8721473; doi:10.1186/s12877-021-02702-z)
Supplement: Supplementary file 1 — Additional file 1. Interview guides for older patients with COPD and for HCPs. [file 12877_2021_2702_MOESM1_ESM.docx]

## Interview guides for older patients with COPD and for HCPs.

| Interview guide for older patients with COPD |
| --- |
| 1. What do you think about engaging in remote CDM for COPD or seeking, finding, understanding, appraising, communicating, applying and creating online health information related to CDM for COPD? (Specially, when it comes to using PeR, what do you think about …) ^a^  What would you think if you saw someone deal with health problems by cellphone or computer or other electronic devices?  2. Could you please share your experience of engaging in remote CDM for COPD or seeking, finding, understanding, appraising, communicating, applying and creating online health information related to CDM for COPD (Specially, when it comes to using PeR, could you please share your experience of …?) ^a^  3. What *has helped/will help* you engage in remote CDM for COPD or seek, find, understand, appraise, communicate, apply and create online health information related to CDM for COPD? (Specially, when it comes to using PeR, what *has helped/will help* you ...?) ^a^  4. What circumstances *has negatively influenced/could negatively influence* your engaging in remote CDM for COPD or your seeking, finding, understanding, appraising, communicating, applying and creating online health information related to CDM for COPD? (Specially, when it comes to using PeR, what circumstances *has negatively influenced/could negatively influence* your ...?) ^a^  5. What do you think it is the *advantages/disadvantages/nothing* for your engaging in remote CDM for COPD or your seeking, finding, understanding, appraising, communicating, applying and creating online health information related to CDM for COPD? (Specially, when it comes to using PeR, what do you think it is the *advantages/disadvantages/nothing* for your...?) ^a^  6. Is there anything else you would like to add? |

^a^ The additional questions in brackets need to be asked for patients who have used PeR, and the ellipsis dots represent the interrogative sentence, which is the same with the interrogative sentence before the brackets.

| Interview guides for HCPs |
| --- |
| 1. What do you think about older patients’ engaging in remote CDM for COPD or seeking, finding, understanding, appraising, communicating, applying and creating online health information related to CDM for COPD? (Specially, when it comes to using PeR, what do you think about...?) ^a^  2. How *did/would* you *deliver remote consultations /nursing/rehabilitation* for older patients with COPD? *Was/Will* there (be) any problems? How *did you/would you like to* solve the problems? (Specially, when it comes to using PeR, how *did* you ...? *Was* …? How *did you* …?) ^a^  3. What makes it *easier/more difficult* for older patients to engage in remote CDM for COPD or to seek, find, understand, appraise, communicate, apply and create online health information related to CDM for COPD? What role do you think you play? (Specially, when it comes to using PeR, what makes it *easier/more difficult*...? What role…?) ^a^  4. Is there anything else you would like to add? |

^a^ The additional questions in brackets need to be asked for HCPs who have used PeR, and the ellipsis dots represent the interrogative sentence, which is the same with the interrogative sentence before the brackets.
